# Inter-predictability of Neuroprognostic Modalities After Cardiac Arrest

**DOI:** 10.7759/cureus.4489

**Published:** 2019-04-17

**Authors:** Tapan Kavi, Masoom Desai, Furkan M Yilmaz, Bhavika Kakadia, Evren Burakgazi-Dalkilic, Gentle S Shrestha

**Affiliations:** 1 Neurology, Cooper Neurological Institute, Cooper University Hospital, Camden, USA; 2 Neurology, Northwestern University Feinberg School of Medicine, Chicago, USA; 3 Critical Care, Tribhuvan University Teaching Hospital, Institute of Medicine, Kathmandu, NPL

**Keywords:** prognostication, cardiac arrest, eeg, ssep, multi-modal approach

## Abstract

Introduction

At present, there is an emphasis on a multi-modal approach to neuro-prognostication after cardiac arrest using clinical examination, neurophysiologic testing, laboratory biomarkers, and radiological studies. However, this necessitates significant resource utilization and can be challenging in under-resourced clinical settings. Hence, we sought to determine the inter-predictability and correlation of prognostic tests performed in patients after cardiac arrest.

Methods

Fifty patients were included through neurophysiology laboratory data for this retrospective study. Clinical, radiological and neurophysiological data were collected. Neurophysiological data were re-evaluated by a board-certified neurophysiologist for the purpose of the study. Chi-square testing was used to evaluate the correlation between different diagnostic modalities.

Results

We found that a non-reactive electroencephalogram (EEG) had a predictive value of 79% for absent bilateral cortical responses (N20) with somatosensory evoked potentials (SSEP). On the other hand, absent bilateral cortical responses N20 had 87% predictive value for a non-reactive EEG. Also, absent cortical responses and non-reactive EEG had predictive values of 78% and 72% for anoxic injury on magnetic resonance imaging (MRI) brain respectively with a non-significant difference on chi-square testing. Individually, absent bilateral N20 SSEP, a non-reactive EEG and anoxic brain injury on MRI studies were highly predictive of poor outcome [modified Rankin scale (mRS) > 4] at hospital discharge.

Conclusion

Neuroprognostication in a post-cardiac arrest setting is often limited by self-fulfilling prophecy. Given the lack of absolute correlation between different modalities used in post-cardiac arrest patients, the value of the multi-modal approach to neuro-prognostication is highlighted by this study.

## Introduction

Neuro-prognostication in patients with an anoxic brain injury after cardiac arrest is challenging, especially with the advent of therapeutic hypothermia (TH) or targeted temperature management (TTM). Current recommendations are to use a multi-modal approach of clinical examination, neurophysiologic testing, biomarkers, and radiological studies [1-3]. Specific results with each of these modalities have been correlated with outcomes in patients with post-anoxic injury and assist in prognosticating patients post-cardiac arrest [1-3]. However, there are no reported studies to our knowledge evaluating the correlation of different modalities used in patients post-cardiac arrest. Electroencephalogram (EEG) and somatosensory evoked potential (SSEP) have been established as robust prognostic markers in cardiac arrest [3]. Non-reactivity to a standard nociceptive, auditory, and visual stimuli and absent N20 SSEP responses post rewarming have been associated with poor neurologic outcomes in post-cardiac arrest patients. In addition, patients with severe anoxic injury on neuro-imaging specifically magnetic resonance imaging have been associated with poor outcomes. Hence, we conducted this study to assess the correlation between different prognostic modalities used in cardiac arrest. We studied if one prognostic modality could predict the results for another modality used in patients post-cardiac arrest. We also correlated the findings obtained on different prognostic modalities with outcomes in these patients. If we could establish a good prediction of outcome with different modalities based on one particular modality, it can help that would spare extensive resource utilization in patients post-cardiac arrest.

## Materials and methods

Patients were identified by retrospective chart review process of the electronic medical record and neurophysiology laboratory studies. A total of 55 successive SSEPs performed in patients with a suspected anoxic brain injury after cardiac arrests were identified between October 2016 and September 2018. Five patients were excluded because of incomplete data. Data collection included patient characteristics such as age, gender, initial cardiac rhythm, initial Glasgow coma scale (GCS). In addition, neuro-prognostic markers such as EEG and SSEP results, magnetic resonance imaging (MRI) brain results, neurological exam, and data on targeted temperature for these patients were obtained. Restricted diffusion or hyperintensity with FLAIR (fluid-attenuated inversion recovery) sequences involving cerebral cortex, or deep nuclei in basal ganglia was used to identify anoxic brain injury on MRI brain. Also, outcome measures such as modified Rankin scale (mRS) at discharge and mortality were elucidated. There was a re-evaluation of previously performed EEG and SSEP studies by a board-certified neurophysiologist for the purpose of this study. Chi-square testing was used as the statistical method to assess the correlation between different diagnostic modalities.

This retrospective study was performed after approval from our Institutional Review Board. Being a retrospective study, no approval from ethical standards committee, institutional or licensing committee, or informed consent from participants was obtained.

## Results

We included 55 patients in the study. Five patients were excluded due to limitations in extracting study results. Majority of the patients, 48 (96%), were subjected to targeted temperature management (TTM) with a goal temperature of 36 degrees Celsius as per institutional practice, as seen in Table 1. At our institution, patients qualify for TTM if their examination after cardiac arrest reveals a GCS score of ≤8 (in accordance with the TTM trial) [4], and presentation is within six hours of cardiac arrest. The remaining patients did not undergo TTM due to presentation outside the window for cooling.

**Table 1:** Baseline characteristics *: Ventricular tachycardia or ventricular fibrillation are shockable rhythms. ^†^: TTM - Targeted temperature management with target of 36 degrees Celsius.

Number	50
Mean age (years)	56
Male %	70
Initial shockable rhythm percent ^*^	40
Percent patients receiving TTM ^†^	96

The median initial GCS score for the patients in our study was 4, as seen in Table 2, with a median motor score of 2. Median GCS score at 72 hours after cardiac arrest improved to 5, and median motor score to 3. About half the patients had pupillary and corneal reactivity at presentation, which increased to 36 (72%) and 33 (66%) for pupillary and corneal reactivity respectively at 72 hours after cardiac arrest. We use a multimodal approach for neuro-prognostication at our institution and prognosticate no earlier than 72 hours after return of spontaneous circulation (ROSC). So, all these patients had SSEP, EEG, and MRI brain performed as part of neuro-prognostication along with the neurological examination.

**Table 2:** Clinical parameters GCS: Glasgow coma scale; CA: Cardiac arrest.

Median Initial GCS	4
Median GCS at 72 hours post CA	5
Median GCS motor score at presentation	2
Median GCS motor score at 72 hours	3
Pupillary Reactivity at presentation	56%
Pupillary reactivity at 72 hours	72%
Corneal Reflex presence at presentation	48%
Corneal Reflex presence at 72 hours	66%
Mortality	74%

Cortical responses (N20s) are assessed for neuro-prognostication and are obtained by stimulation of bilateral median nerves during SSEP evaluation. As per institutional protocol, continuous EEG is obtained on all cardiac arrest patients during the rewarming phase of TTM, unless indicated earlier (to rule out myoclonic status epilepticus or non-convulsive status epilepticus). Rewarming is conducted at a rate of 0.25 degree Celsius rise per hour at our institution. For the patients included in this study, EEG was performed at a median of 38 hours from cardiac arrest. In addition, SSEPs were performed at a median of 84 hours after cardiac arrest (48 hours after rewarming completion). The correlation of results between these two closely related neurophysiologic tests was assessed using a 2 x 2 table as seen in Table 3. We found that a non-reactive EEG (to standard nociceptive, auditory, and visual stimuli) was not always predictive of absent cortical responses (N20) on SSEP with a predictive value of 79%. Correspondingly, absent cortical responses on SSEP were predictive of non-reactivity on EEG in 87% of the cases. The predictive value of good prognostic sign on SSEP (intact cortical response) for good prognostic sign on EEG (reactive EEG) was very poor, with a predictive value of 18%. This suggests that both these tests have better inter-predictability for poor prognostic signs than good prognostic signs.

**Table 3:** SSEP and EEG inter-predictability SSEP-N20: Somatosensory evoked potential N20; EEG: Electroencephalography; PV: Predictive value.

	Reactive EEG	Non-reactive EEG	PV
SSEP- N20 present	2	9	2/11 (18%)
SSEP- N20 absent	5	34	34/39 (87%)

We also assessed the predictability of SSEP and EEG poor prognostic findings for evidence of anoxic brain injury on neuroimaging studies. As shown in Table 4, absent cortical responses and non-reactive EEG had predictive values of 85% and 81% for anoxic injury on neuroimaging respectively with a non-significant difference between the two tests (EEG and SSEP) to predict anoxic injury on chi-square testing. Moreover, the correlation of good prognostic signs (present cortical responses, reactive EEG and absence of anoxic injury on neuroimaging) amongst each other was very poor in this study population.

**Table 4:** SSEP, EEG and neuroimaging inter-predictability SSEP-N20: Somatosensory evoked potential N20; MRI: Magnetic resonance imaging; EEG: Electroencephalography; PV: Predictive value.

	MRI- anoxic injury absent	MRI- anoxic injury present	PV		MRI- anoxic injury absent	MRI- anoxic injury present	PV
SSEP- N20 present	4	7	4/11 (36%)	EEG- reactive	2	5	2/7 (29%)
SSEP- N20 absent	6	33	33/39 (85%)	EEG non-reactive	8	35	35/43 (81%)

We assessed the predictability of SSEP, neuroimaging, and EEG for outcomes using the modified Rankin Scale at hospital discharge. A cut-off of mRS 4 at hospital discharge was used to consider a good outcome. As seen in Table 5, absent cortical responses (100%) and non-reactive EEG (100%) had very high predictive value for poor outcome (mRS > 4). Evidence of anoxic injury on MRI brain also had a very high predictive value for poor outcome (97%). Moreover, the predictive value of SSEP, EEG or MRI good prognostic signs for a good outcome was very poor; 27%, 43%, and 20% respectively in this study population. The mortality rate in our study was high at 37/50 (74%), which reflects a high prevalence of severe anoxic brain injury in the study population.

**Table 5:** SSEP, EEG, MRI and outcome prediction SSEP-N20: Somatosensory evoked potential N20; mRS: Modified Rankin Scale; MRI: Magnetic resonance imaging; EEG: Electroencephalography; PV: Predictive value.

	mRS < or = 4	mRS > 4	PV		mRS < or = 4	mRS > 4	PV		mRS < or = 4	mRS > 4	PV
SSEP-N20 present	3	8	3/11 (27%)	MRI- anoxic injury absent	2	8	2/10 (20%)	EEG reactive	3	4	3/7 (43%)
SSEP- N20 absent	0	39	39/39 (100%)	MRI- anoxic injury present	1	39	39/40 (97%)	EEG non-reactive	0	43	43/43 (100%)

The preliminary results of this study were presented at the Neurocritical Care Society (NCS) 16th Annual Meeting in 2018. (Abstract: Yilmaz FM, Kakadia B, Kouch M, Hunter K, Burakgazi-Dalkilic E, Kavi T: Inter-predictability of Neuroprognostic Modalities after Cardiac Arrest. Presented at the Neurocritical Care Society (NCS) 16th Annual Meeting, Boca Raton, FL, USA; September 25-28, 2018.)

## Discussion

Neurologic prognostication after cardiac arrest is a crucial part of the management of patients post-cardiac arrest with significant consequences. Modulation of brain injury and slowed metabolism of sedative medications leads to delayed recovery of brain function and findings on neurologic examination [5]. For this reason, neurological exam and prognostication early on after resuscitation from cardiac arrest can be unreliable and waiting at least 72 hours after ROSC is recommended [1-3]. Recommendations also include using a multimodal approach to neurologic prognostication by using clinical examination, neurophysiological testing, radiological testing and laboratory biomarkers [1-3]. The ideal neuro-prognostic method or tool should have a negligible false positive rate (FPR) and should approach zero. This would ensure avoidance of premature discontinuation of life-supportive measures for patients who have a chance of meaningful recovery in the future.

In the setting of therapeutic hypothermia, the clinical examination remains a robust tool for neuro-prognostication. The absence of bilateral pupillary reflex at 72 hours after cardiac arrest has an FPR of 0.5% for prediction of poor neurologic outcome [6-9]. On the other hand, corneal reflexes, motor response to pain and early post-anoxic myoclonus have a higher FPR and lower utility for neuro-prognostication [6-12]. When malignant features (burst suppression pattern, myoclonic status epilepticus) on the EEG findings are seen along with myoclonus, the predictability for poor prognosis is much higher [13]. Neurophysiologic testing with EEG and SSEP has retained high utility for prognostication. EEG abnormalities such as suppressed or discontinuous background, and burst suppression have an FPR close to zero for prognosis, but the absence of reactivity or epileptiform discharges have higher FPR [14-16]. In addition, bilateral absence of cortical responses on SSEP testing has been shown to have FPR close to zero for neuro-prognostication in this patient cohort [17]. Computed tomography (CT) and MRI have less data on prognostication as compared to clinical or neurophysiologic testing, however loss of gray-white differentiation on CT is a very reliable predictor (FPR of zero in multiple studies) [18,19]. Serum Neuron-Specific Enolase has lower utility for prognostication after TTM, as even higher cut-offs have been reported with good neurologic outcomes [20-22].

However, performing all these prognostic studies can lead to considerable resource utilization and imposes a significant financial burden in a resource-limited clinical setting. If studies have a high correlation for poor prognostic markers, selected tests could be utilized for prognostication. SSEP and EEG, being closely related neurophysiological tests, are particularly interesting in this regard. SSEP differs from EEG in that it is an evaluation of afferent response, while EEG is an evaluation of efferent response-generation of neuronal signals. EEG reactivity in comatose survivors of CA seems to be a stronger predictor of good prognosis than the presence of cortical responses on SSEP testing [23].

Our study found that there was not an absolute correlation between markers of poor outcome when assessed by using SSEP, EEG, and MRI. The reason for this can be explained by the fact that each of these modalities has specific limitations. For example, reactivity on the EEG as a prognostic marker can be limited by the applied stimulus, the inter-rater reliability of reactivity on EEG and at times confounding factors like sedation usage in this patient population. SSEPs, while a robust tool for prognostication, are still not absolute markers of poor outcomes and carry limitations of being operator dependent. Neuro-imaging for evaluation of anoxic brain injury has limited prognostic value by itself in this patient cohort. So, this study re-affirms the value of multi-modal approach in neuro-prognostication of patients post-cardiac arrest. The study also confirms previous findings that absent cortical responses on SSEP are a robust tool for neuro-prognostication in patients post-cardiac arrest.

Our study has certain limitations. One of the main limitations of this study is the withdrawal of life support, reflecting the self-fulfilling prophecy of the intensivists taking care of these patients. The small sample size is also a limitation of the study. Also, the study population is biased towards those with severe anoxic brain injury. SSEP and other prognostic tests were obtained in those with a poor neurologic examination after cardiac arrest, and hence the predictive value seen in this study is limited in this population and not applicable to all survivors of cardiac arrest. This can also be the reason for the poor predictive value of these tests for a good prognosis.

It would be interesting to assess if a model based on a combination of the different diagnostic modalities and clinical examination predicts outcomes post-cardiac arrest more robustly than a single diagnostic study by itself.

## Conclusions

The study shows that there is not an absolute correlation between different prognostic markers when it comes to predicting poor outcomes. Given the limitations associated with withdrawal of life support in neuro-prognostication after cardiac arrest and the lack of absolute predictability of one test for the other, the study reaffirms the value of a multi-modal approach to prognostication after cardiac arrest.
